# Quality of life and associated factors among patients with cancer receiving chemotherapy at Dessie Comprehensive Specialized Hospital, North-East Ethiopia: a cross-sectional study

**DOI:** 10.3389/fonc.2023.1288166

**Published:** 2024-01-08

**Authors:** Ewunetie Mekashaw Bayked, Mekdes Getachew Yimam, Zemen Mengesha Yalew, Husien Nurahmed Toleha, Segenet Zewdie

**Affiliations:** ^1^ Department of Pharmacy, College of Medicine and Health Sciences (CMHS), Wollo University, Dessie, Ethiopia; ^2^ Department of Oncology, Dessie Comprehensive Specialized Hospital (DCSH), Dessie, Ethiopia; ^3^ Department of Comprehensive Nursing, College of Medicine and Health Sciences (CMHS), Wollo University, Dessie, Ethiopia; ^4^ Department of Pharmacy, College of Medicine and Health Science, Injibara University, Injibara, Ethiopia

**Keywords:** cancer, patients, chemotherapy, quality of life, associated factors

## Abstract

**Background:**

Cancer is a major public health problem around the world. Cancer by itself and its treatment modalities affect the quality of life (QoL) of patients with it. However, there were a paucity of studies about the QoL of patients receiving chemotherapeutic treatment in Ethiopia. This study was aimed at addressing such a gap. Accordingly, we investigated QoL and associated factors among cancer patients receiving chemotherapy at Dessie Comprehensive Specialized Hospital (DCSH), North East Ethiopia, in 2023.

**Methods:**

We employed a cross-sectional study from April 1 to May 30, 2023. The data was collected using the European Organization for Research and Treatment of Cancer Core QoL Questionnaire, version 3.0 (EORTC QLQ-C30). The data was entered and cleaned using EpiData version 4.6 and exported to Statistical Package for Social Sciences (SPSS) version 27 for analysis. The association between the dependent and independent variables was determined using Odds Ratios (ORs) at a p value < 0.05 with a 95% CI.

**Results:**

Data was collected from 394 patients. Their mean summary QoL score was 36.3 ± 9.0. About 39.3% demonstrated a good QoL summary score, whereas 60.7% were impacted by symptoms. A good functional QoL score was observed in 42.6% of the participants. About 54.8% and 31.7% reported good overall health status and good overall QoL, respectively. The most impacted functional domain was social functioning, affecting 64.5% of participants. The most common symptom was diarrhea, affecting 65.5% of the participants. Secondary school education level (Adjusted Odds Ratio-AOR = 3.16, 95% CI: 1.14-8.81), diploma and above education level (AOR = 4.90, 95% CI: 1.29-18.62), and urban residency (AOR = 1.74, 95% CI: 1.07-2.82) had a significant positive association with QoL, while being a civil servant (AOR = 0.13, 95% CI: 0.04-0.49), having stage III cancer (AOR = 0.14, 95% CI: 0.05-0.39), and stage IV cancer (AOR = 0.16, 95% 0.06-0.44) had a significant negative association with it.

**Conclusion:**

The QoL for cancer patients undergoing chemotherapy was significantly low and associated with their level of education, occupational status, area of residence, and stage of cancer. Incorporating psychosocial support is thus crucial in their treatment plans.

## Introduction

The World Health Organization (WHO) defines cancer as a broad term encompassing a variety of diseases that can impact any part of the body (1). It is characterized by the uncontrolled proliferation and spread of certain body cells to other areas (2). Cancer is a multi-stage process involving normal cells transforming into tumors, influenced by genetics, external agents, and infections (1). Normal cells can transform into cancer cells due to abnormal alterations known as hyperplasia and dysplasia (2).

Age-related cancer incidence increases due to increased risks and a decline in cellular repair processes. Tobacco, alcohol, unhealthy diets, a lack of physical activity, and air pollution are other major contributors to cancer. Chronic infections such as human papillomavirus (HPV), hepatitis B and C, and Epstein-Barr virus heighten the risk of developing cancer, particularly in low- and middle-income countries. Moreover, human immunodeficiency virus (HIV) infection elevates the risk of cervical cancer and increases the likelihood of Kaposi’s sarcoma (1).

In 2020, 19.3 million new cancer cases and 10.0 million deaths were reported globally (3). Nearly 18.1 million new cases of cancer were reported worldwide in 2020 (4). Cancer is the second leading global cause of death, with 70% of deaths in low- and middle-income countries (LMICs) (5). In recent years, the incidence of new cancer cases in the LMICs has increased significantly due to a growing population and exposure to risk factors (4). Cancer is an escalating public health issue in low-income nations, particularly in Ethiopia. Based on a systematic review, it was estimated that there were 53,560 new cancer cases and 39,480 cancer-related deaths in this country in 2019 (6).

**Figure 1:**
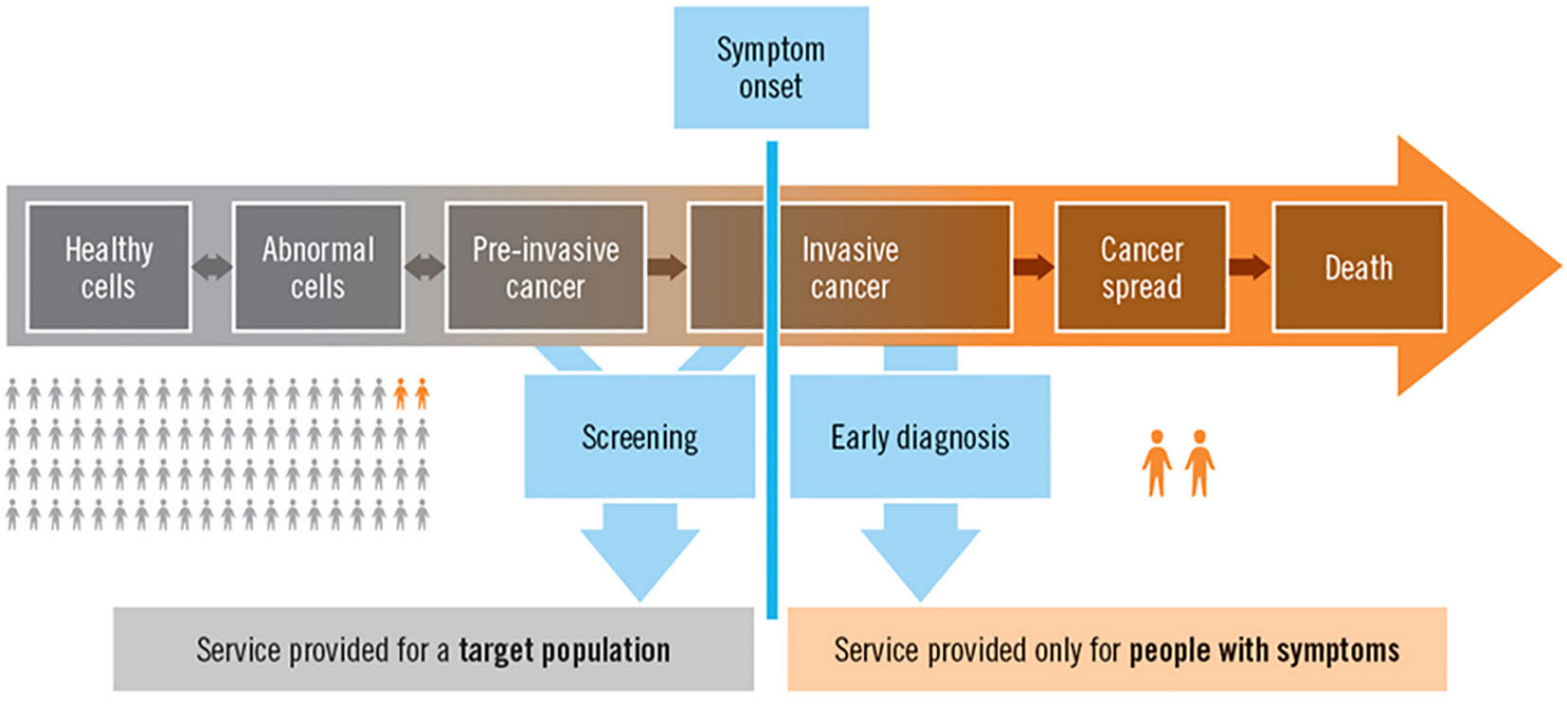
The natural history of cancer, surveillance, screening, diagnosis, and service provision at the population and individual levels (8).

Cancer incidence and mortality rates in Africa are rising; however, their geographic distribution and determinants are not well understood. One of the major opportunities for incidence and mortality surveillance is to provide information to developing countries, especially in Africa, where many cancer registries are coming online (7). Cancer surveillance can discuss the number of patients and their condition at the population level. However, these efforts should also consider the needs of individual patients, which is the focus of this paper, **Figure 1** (8).

Cancer is a severe disease that affects the body, mind, and spirit of patients (9). It significantly affects QoL, such as physical and psychological health, social interactions, and overall bodily functions (10). WHO defines QoL as individuals’ perception of their position in life, influenced by culture, values, goals, expectations, standards, and concerns (11). QoL is a crucial goal in contemporary healthcare (12), particularly in chronic illness treatment (13). Cancer and its chemotherapy negatively impact individual well-being (14, 15), making QoL the primary objective for survival (16).

Assessing QoL is crucial for cancer patients’ physical, emotional, and social well-being (17). Therefore, a comprehensive approach to cancer prevention, early detection, treatment, and pain management is essential for 21st-century cancer control. Cancer continues to be a leading cause of death in LMICs, including Ethiopia, due to inadequate interventions such as limited access to vaccinations and screenings, late diagnoses, inadequate treatment options, a lack of pain relief, and insufficient psycho-social support (5).

The QoL of cancer patients in Ethiopia is low (18), attributed to a lack of awareness, education, and delayed diagnosis, resulting in advanced stages and a poorer prognosis (19). Ethiopia faces a shortage of healthcare professionals, causing delayed treatment (20). It also faces financial barriers to cancer care, including high costs, limited access, and a lack of insurance coverage (21). On the other hand, cancer affects QoL through physical symptoms, emotional distress, and chemotherapy side effects, requiring healthcare providers to collaborate and improve patient well-being (22). These issues may also intensify based on the patients’ sociodemographic characteristics, the nature and stage of their disease, its symptoms, and their health insurance status. Hence, the objective of this study was to ascertain the QoL of patients with cancer receiving chemotherapy and associated factors at Dessie Comprehensive Specialized Hospital (DCSH) in 2023.

## Methods

### Study period, design, and setting

A cross-sectional study was conducted at DCSH from April to June 2023. DCSH is one of four cancer treatment centers in the Amhara region of Ethiopia; the others being Gondar Comprehensive Specialized Hospital (GCSH), Felegehiwot Comprehensive Specialized Hospital (FCSH), and Tibebegion Comprehensive Specialized Hospital (TCSH). DCSH is situated in Dessie City Administration, located 401 kilometers northeast of Addis Ababa (23). The largest and highest tertiary hospital in Ethiopia’s northeastern region, with the broadest catchment area, is DCSH. It serves South Wollo, North Wollo, Waghimra, the Oromia Special Zones, parts of the North Shoa Zone in the Amhara region, as well as the Afar and South Tigray regions (24). In addition to its regular operations, it offers 13 distinct specialty services. The hospital has a capacity exceeding 500 beds and employs a total of 925 individuals, including 616 medical professionals and 309 administrative staff (25).

Currently, over 10 million people are receiving comprehensive, interconnected services from the hospital. The oncology unit has been treating patients with cancer since it began operations on December 12, 2017. Initially, the department started with 10 beds and a team consisting of five nurses, three general practitioners, three pharmacists, and one clinical oncologist. On average, the hospital serves around 450 cancer patients undergoing chemotherapy each month.

### Participants and sample

The study encompassed adult cancer patients undergoing chemotherapy, however, those with severe illnesses were excluded. Patients who had received at least three cycles of chemotherapy were included in the study. Those who were seriously ill were excluded. The sample size was calculated using the single population proportion formula. Since there were no previous studies on the QoL of patients with cancer receiving chemotherapy in Ethiopia, we assumed a proportion of 50% of participants had good QoL with a 5% margin of error and a 95% CI (Equation 1).


(1) $$n=\frac{Z^{2}\;\left(p\right)\left(1-p\right)}{d^{2}}\;=\frac{{\left(1.96\right)}^{2}\;\left(0.5\right)\left(0.5\right)}{{\left(0.05\right)}^{2}}\;=384$$

The total sample size was determined to be 403 by adding 5% non-response (384 × 0.05 = 19.2). Though the size of the population was less than 10,000, the investigators did not use a correction formula in order to increase the sample size to obtain reliable representative data.

A systematic random sampling technique was employed to select the participants for the interview, using their registration book as a sampling frame. The oncology ward of the hospital catered to 450 cancer patients every month. Given the sample size and the number of patients served monthly, participants were interviewed at every K^th^ interval, which is approximately 1 (450 ÷ 403). Consequently, as the study was conducted over a month during working days only, each day’s sample consisted of approximately 20 participants (403 ÷ 20). On average, the hospital attended to 23 cancer patients daily, excluding weekends. Since the interval was 1, the participants were interviewed every day in a consecutive manner until we obtained the required sample size over the course of a month.

### Instrument and data collection

The data were collected using the EORTC QLQ-C30 version 3 (26), a 30-item tool designed for all types of cancer. Version 3.0 is currently the standard version of the QLQ-C30 and should be used for all new studies unless investigators wish to maintain compatibility with previous studies that used an earlier version of the QLQ-C30. It consists of multi-item scales and single-item measures, including functional, symptom, and global scales, with no item appearing on more than one scale. The QLQ-C30 version 3.0 uses four-point scales for items 1 to 28, where “not at all” is rated as 1, “a little” as 2, “quite a bit” as 3, and “very much” as 4. Furthermore, this tool includes two global items, which use seven-point scales, to test whether the summary score is consistent with the scores of these overall items. Fayers et al. (2001) recommended using version 3.0 for new studies (27), as it is designed to measure cancer patients’ physical, psychological, and social functions (28). Using interviewer-administered techniques, the data collection was conducted by three trained and experienced BSc nurse professionals.

### Data processing and analysis

The data were entered, categorized, and refined using EpiData 4.6, then analyzed with SPSS-27. Descriptive statistics were presented through frequency tables. We also conducted a logistic regression analysis to determine the association between the dependent variable (QoL) and the independent variables (socio-demographic and disease characteristics). First, we used a bivariable logistic regression model to determine the relationship between each independent variable and the outcome variable (QoL). Second, we included all variables in the multivariable model to assess how one variable was affected (adjusted) by others when they were run simultaneously. During the multivariable analysis, independent variables with a p-value of less than 0.05 were considered significantly associated with the outcome variable, i.e., QoL.

The data processing, analysis, and interpretations adhered to the guidelines of the EORTC QLQ-C30 Scoring Manual developed by Fayers et al. (2001). As per Fayers et al. (2001), all publications pertaining to the QLQ should employ the scoring procedures outlined in this manual. The manual instructs researchers to initially categorize each item into functional, symptom, and global health scales, then compute the raw mean scores for each scale category. The technique for calculating these raw scores is given in Equation 2 (27), where “I” represents a single item and “n” stands for the number of items within a scale.


(2) $$Raw\;scale=\;\frac{\left(I_{1}+I_{2}+\ldots +I_{n}\right)}{n}$$

On the raw scale, an increase in value could lead to increased dysfunctionality and symptom burdens. However, on a global scale, a higher score indicates improved overall health status, or QoL. Subsequently, all scales and single-item measures were standardized and linearly transformed into a score range of 0 to 100. A high score on the scale signifies a higher response level. In other words, a higher score denotes either an enhanced level of functioning or an escalated level of symptoms. The method for calculating the score is depicted in Equation 3 (27).


(3) $$Score=\;\frac{Raw\;scale-1}{Range}$$

The range, which is the difference between the maximum and minimum scale points, is 3 for items 1 to 28 and 6 for global items. As shown in Equation 4, for functional scales (FS), subtract the score (Equation 3) from 1 and multiply by 100 to simplify interpretation (27).


(4) $$Linearly\;trasnsformed\;functional\;scale=(1-\left(\frac{Raw\;scale-1}{Range}\right)\times 100$$

For the symptoms and global scales, the score, as indicated in Equation 3, was multiplied by 100 to derive the mean percentage values. These values ranged from 0 to 100, as demonstrated in Equation 5 (27).


(5) $$Linearly\;transformed\;symptom\;or\;global\;scales=\left(\frac{Raw\;scale-1}{Range}\right)\times 100$$

A high score on a functional scale indicates a healthy level of functioning. Similarly, a high score on the global health status, or QoL, signifies a high overall health status, or a high QoL. However, a high score on a symptom scale suggests a high level of symptoms or problems (27).

The mean summary score for QLQ-C30 should be computed as the average of the combined 13 scales, excluding global scales and financial impact. A higher score signifies better QoL (29). Thus, as outlined in Equation 6, the mean summary score was calculated using the following command: (Physical Functioning + Role Functioning + Social Functioning + Emotional Functioning + Cognitive Functioning + 100 - Fatigue + 100 - Pain + 100 - Nausea and Vomiting + 100 - Dyspnea + 100 - Sleep Disturbances + 100 - Appetite Loss + 100 - Constipation + 100 - Diarrhea) / 13.


(6) $$\begin{array}{c} Mean\;QLQ_{C30}\;summary\;score\\ =\;\frac{\sum \left(Functional\;scales\right)\;+\;\sum \left(100-symptom\;scales\right)}{13} \end{array}$$

Finally, to ascertain the QoL score, for functional, global (overall), and mean summary QoL, the score above the mean was labeled “good QoL” and the score below the mean “poor QoL,” while for the symptom scales, the score below the mean was labeled “good QoL” and the score above the mean “poor QoL”.

### Data quality control

We used the standardized EORTC QLQ-C30 version 3 for data collection (26). The Hosmer and Lemeshow Test was conducted to assess the model fitness of the binary logistic regression, which proved to be non-significant (p = 0.457). Each questionnaire was meticulously checked for completeness during the data collection process. Incomplete questionnaires or those with missing data or errors were rejected. The scoring procedures adhered to a standardized guideline, ensuring accuracy and consistency (27). All collected data were securely stored and maintained.

## Results

### Socio-demographic characteristics

The study was conducted on 394 participants, with a response rate of 97.8%. The mean (SD) age of the participants was 46.62 ( ± 15.47) years. Their median age was 45.7 years, with an age range of 69 years. The interquartile range (IQR) of their age was 21.61 years. The majority of the participants (126, or 32.0%) were in the age range of 41–55 years. Most of them were female (62.4%). Two hundred ninety-one (73.9%) of the respondents were married. Two hundred and two (51.3%) of them were illiterate. Regarding occupational status, most of them were farmers and housewives, accounting for 138 (35%) and 134 (34%) of the participants, respectively. More than 55% of them were urban residents. More than half (51.3%) of them were followers of orthodox Christianity. The dominant ethnic group was Amhara, constituting 302 (76.6%) of the respondents (**Table 1**).

**Table 1:** Socio-demographic characteristics of patients with cancer receiving chemotherapy at DCSH (n = 394), North-East Ethiopia, 2023.

Variable		Frequency	Percentage
**Age (years)**	11-25 years	35	8.9
	26-40 years	114	28.9
	41-55 years	126	32.0
	56-70 years	92	23.4
	71-85 years	27	6.9
**Sex**	Male	148	37.6
	Female	246	62.4
**Marital status**	Married	291	73.9
	Single	68	17.3
	Widowed	17	4.3
	Divorced	18	4.6
**Educational level**	Unable to write and read	202	51.3
	Primary school	132	33.5
	Secondary school	35	8.9
	Diploma and above	25	6.3
**Occupational status**	Farmer	138	35.0
	House wife	134	34.0
	Merchant	54	13.7
	Civil servant	37	9.4
	Other	31	7.9
**Residency**	Rural	177	44.9
	Urban	217	55.1
**Religion**	Orthodox Christianity	202	51.3
	Muslim	175	44.4
	Protestant	17	4.3
**Ethnicity**	Amhara	302	76.6
	Oromo	49	12.4
	Afar	33	8.4
	Other	10	2.5

### Disease and health insurance conditions

Most (262, or 66.5%) of the patients had health insurance to offset healthcare costs. Among all of the participants, breast cancer was found to be the most common type of cancer, accounting for 40.1% of all cancer cases. The majority (40.6%) of patients had stage IV cancer (**Table 2**).

**Table 2:** Disease and health insurance conditions of patients with cancer receiving chemotherapy at DCSH (n = 394), North-East Ethiopia, 2023.

Variable		Frequency	Percentage
**Health insurance status**	Uninsured	132	33.5
	Insured	262	66.5
**Type of cancer**	Breast cancer	158	40.1
	Cervical cancer	73	18.5
	Colon cancer	32	8.1
	Liver cancer	51	12.9
	Non-Hodgkin’s Lymphoma (NHL)	61	15.5
	Other	19	4.8
**Stage of cancer**	Stage I	33	8.4
	Stage II	80	20.3
	Stage III	121	30.7
	Stage IV	160	40.6

### Quality of life score

The unadjusted (raw) scores revealed average scores of 2.93 and 2.99 for the functional and symptom scales respectively. The highest average score among the functional scales was 3.36, suggesting patients experienced significant dysfunction in social activities. In terms of symptoms, dyspnea had the highest average score at 3.50. Both overall health status and QoL scores were notably low (**Table 3**).

**Table 3:** Raw and transformed mean scores of the QoL scales among cancer patients undertaking chemotherapy at DCSH (n = 394), North-East Ethiopia, 2023.

Scales	Raw mean ± SD	Transformed mean ± SD
**Overall health status**	2.92 ± 1.52	32.0 ± 25.3
**Overall QoL**	3.14 ± 1.35	35.7 ± 22.4
**Functional scales**	**2.93 ± 0.37**	**37.0 ± 10.42**
Physical functioning (PF)	2.67 ± 0.39	44.2 ± 13.2
Role functioning (RF)	2.76 ± 0.70	41.3 ± 23.2
Emotional functioning (EF)	2.60 ± 0.45	53.2 ± 15.1
Cognitive functioning (CF)	3.25 ± 0.79	25.0 ± 26.3
Social functioning (SF)	3.36 ± 0.75	21.5 ± 25.0
**Symptom scales**	**2.99 ± 0.34**	**66.2 ± 11.43**
Fatigue (FA)	2.84 ± 0.48	61.4 ± 16.1
Nausea and vomiting (NV)	3.27 ± 0.62	75.6 ± 20.7
Pain (PA)	3.15 ± 0.69	71.7 ± 23.0
Dyspnea (DY)	3.50 ± 0.72	83.3 ± 23.9
Insomnia (SL)	3.39 ± 0.75	79.6 ± 25.0
Appetite loss (AP)	3.07 ± 0.85	69.0 ± 28.2
Constipation (CO)	2.20 ± 0.81	40.1 ± 27.1
Diarrhea (DI)	1.98 ± 0.88	32.8 ± 29.4
Financial difficulties (FI)	3.48 ± 0.76	82.6 ± 25.2
**Mean summary QoL score**		**36.3 ± 9.0**

The bold figures represent the aggregate mean values.

After transforming the raw mean scores of the scales, the functional and symptom scales had mean scores of 37.03 and 66.24, respectively. The highest mean scores were observed for physical functioning and symptom experience, at 53.2 and 83.3, respectively. The overall health status and overall QoL had mean scores of 32.0 and 35.7, respectively. The mean summary QoL score was 36.3 (**Table 3**).

**Table 4** revealed that only 39.3% of participants had a good summary QoL score. Concerning the symptom scales, 60.7% of the respondents demonstrated poor QoL. Participants with a good functional QoL score constituted 42.6%. Additionally, 54.8% and 31.7% of participants had good overall health status and overall QoL, respectively. The most impacted functional scale was social functioning, with 64.5% of participants exhibiting poor performance in social activities. The most prevalent symptom was diarrhea, affecting 65.5% of participants.

**Table 4:** The mean QoL summary, overall, functional, and symptom scores among cancer patients undertaking chemotherapy (n = 394), North-East Ethiopia, 2023.

Scales	Mean ± SD	Good QoL, No (%)	Poor QoL, No (%)
**Overall health status**	32.0 ± 25.3	216 (54.8)	178 (45.2)
**Overall QoL**	35.7 ± 22.4	125 (31.7)	269 (68.3)
**Functional scales**	**37.0 ± 10.42**	**168 (42.6)**	**226 (57.4)**
Physical functioning (PF)	44.2 ± 13.2	194 (49.2)	200 (50.8)
Role functioning (RF)	41.3 ± 23.2	185 (47.0)	209 (53.0)
Emotional functioning (EF)	53.2 ± 15.1	199 (50.5)	195 (49.5)
Cognitive functioning (CF)	25.0 ± 26.3	167 (42.4)	227 (57.6)
Social functioning (SF)	21.5 ± 25.0	140 (35.5)	254 (64.5)
**Symptom scales**	**66.2 ± 11.43**	**155 (39.3)**	**239 (60.7)**
Fatigue (FA)	61.4 ± 16.1	166 (42.1)	228 (57.9)
Nausea and vomiting (NV)	75.6 ± 20.7	157 (39.8)	237 (60.2)
Pain (PA)	71.7 ± 23.0	184 (46.7)	210 (53.3)
Dyspnea (DY)	83.3 ± 23.9	153 (38.8)	241 (61.2)
Insomnia (SL)	79.6 ± 25.0	187 (47.5)	207 (52.5)
Appetite loss (AP)	69.0 ± 28.2	259 (65.7)	135 (34.3)
Constipation (CO)	40.1 ± 27.1	240 (60.9)	154 (39.1)
Diarrhea (DI)	32.8 ± 29.4	136 (34.5)	258 (65.5)
Financial difficulties (FI)	82.6 ± 25.2	153 (38.8)	241 (61.2)
**Mean summary QoL score**	**36.3 ± 9.0**	**155 (39.3)**	**239 (60.7)**

The bold figures represent the aggregate mean values.

### Factors affecting quality of life

The QoL of the participants was found to be affected by sociodemographic variables such as level of education, occupational status, residency, and stage of cancer (**Table 5**). Patients who had secondary school and higher (diploma and above) level education were 3.16 times (AOR = 3.16, 95% CI: 1.14–8.81) and 4.90 times (AOR = 4.90, 95% CI: 1.29–18.62) more likely to have better QoL, respectively, than those who were unable to write and read. Those patients who were civil servants were 87% less likely (AOR = 0.13, 95% CI: 0.04–0.49) to have a good QoL than those who were farmers. The patients who were living in urban areas were 1.74 times more likely (AOR = 1.74, 95% CI: 1.07–2.82) to have a better QoL than those who were living in rural areas. Regarding the stage of cancer, those participants who had stage III and IV cancer were 86% less likely (AOR = 0.14, 95% CI: 0.05-0.39) and 84% less likely (AOR = 0.16, 95% CI: 0.06-0.44), respectively, to have good QoL than those who had stage I cancer.

**Table 5:** The factors affecting the mean summary QoL of patients with cancer receiving chemotherapy at DCSH (n = 394), North-East Ethiopia, 2023.

Variables		No (%)	COR (95% CI)	AOR (95% CI)	P value
**Age (years)**	11-25 years	35 (8.9)	1	1	
	26-40 years	114 (28.9)	0.74 [0.35,1.58]	1.37 [0.53,3.55]	0.521
	41-55 years	126 (32.0)	0.56 [0.26,1.20]	1.40 [0.52,3.74]	0.504
	56-70 years	92 (23.4)	0.43 [0.2,0.96]	1.06 [0.39,2.92]	0.906
	71-85 years	27 (6.9)	0.65 [0.24,1.79]	2.86 [0.81,10.10]	0.102
**Sex**	Male	148 (37.6)	1	1	
	Female	246 (62.4)	1.06 [0.70,1.61]	1.15 [0.66,2.00]	0.615
**Marital status**	Married	291 (73.9)	1	1	
	Single	68 (17.3)	1.80 [1.06,3.06]	1.37 [0.68,2.76]	0.372
	Widowed	17 (4.3)	0.21 [0.05,0.95]	0.21 [0.04,1.05]	0.057
	Divorced	18 (4.6)	0.61 [0.21,1.77]	0.72 [0.23,2.27]	0.569
**Educational level**	Unable to write and read	202 (51.3)	1	1	
	Primary school	132 (33.5)	1.35 [0.86,2.12]	1.25 [0.72,2.14]	0.427
	Secondary school	35 (8.9)	2.00 [0.97,4.12]	3.16 [1.14,8.81] *	0.027
	Diploma and above	25 (6.3)	1.74 [0.75,4.02]	4.90 [1.29,18.62] *	0.020
**Occupational status**	Farmer	138 (35.0)	1	1	
	House wife	134 (34.0)	1.26 [0.78,2.05]	0.84 [0.46,1.52]	0.561
	Merchant	54 (13.7)	1.09 [0.57,2.07]	0.57 [0.26,1.28]	0.173
	Civil servant	37 (9.4)	0.63 [0.28,1.41]	0.13 [0.04,0.49] **	0.003
	Other	31 (7.9)	1.82 [0.83,3.99]	0.59 [0.20,1.70]	0.330
**Residency**	Rural	177 (44.9)	1	1	
	Urban	217 (55.1)	1.59 [1.05,2.39]	1.74 [1.07,2.82] *	0.026
**Religion**	Orthodox Christianity	202 (51.3)	1	1	
	Muslim	175 (44.4)	1.04 [0.69,1.57]	0.93 [0.58,1.50]	0.763
	Protestant	17 (4.3)	0.47 [0.15,1.49]	0.47 [0.13,1.68]	0.247
**Ethnicity**	Amhara	302 (76.6)	1	1	
	Oromo	49 (12.4)	0.81 [0.43,1.52]	0.79 [0.39,1.58]	0.498
	Afar	33 (8.4)	1.12 [0.54,2.31]	1.07 [0.48,2.40]	0.872
	Other	10 (2.5)	1.01 [0.28,3.66]	1.58 [0.34,7.27]	0.555
**Health insurance status**	Uninsured	132 (33.5)	1	1	
	Insured	262 (66.5)	0.82 [0.54,1.26]	0.73 [0.44,1.20]	0.214
**Type of cancer**	Breast cancer	158 (40.1)	1	1	
	Cervical cancer	73 (18.5)	1.05 [0.59,1.85]	1.21 [0.63,2.29]	0.567
	Colon cancer	32 (8.1)	0.83 [0.38,1.85]	1.16 [0.45,2.99]	0.761
	Liver cancer	51 (12.9)	0.49 [0.24,1.01]	0.62 [0.26,1.44]	0.265
	NHL	61 (15.5)	1.87 [1.03,3.40]	0.92 [0.43,1.99]	0.834
	Other	19 (4.8)	1.43 [0.55,3.72]	1.17 [0.37,3.74]	0.791
**Stage of cancer**	Stage I	33 (8.4)	1	1	
	Stage II	80 (20.3)	0.51 [0.21,1.23]	0.49 [0.19,1.30]	0.152
	Stage III	121 (30.7)	0.16 [0.07,0.38]	0.14 [0.05,0.39] ***	<0.001
	Stage IV	160 (40.6)	0.17 [0.07,0.38]	0.16 [0.06,0.44] ***	<0.001

*Significant at a p value <0.05; **Significant at a p value <0.01; ***Significant at a p value <0.001.

AOR, Adjusted Odds Ratio; COR, Crude Odds Ratio.

## Discussion

The study revealed that the mean score of the mean summary QoL score was found to be 36.3. This mean summary QoL score was approximately equivalent to the overall mean QoL score, which was 35.7. The overall mean health status (32.0), however, was less than the mean summary QoL and the overall QoL scores. This finding was much lower than a similar study conducted in Gondar, Ethiopia, which reported that the mean QoL of patients with cancer was 52.7 with a standard deviation of 20.1 (18). A similar recent study conducted in the cancer centers of Amhara Regional State also reported a higher average QoL score of 44.32 (19). The mean score of the QoL of patients with cancer in this study was also much lower than the national pooled result of various studies in Ethiopia, which was 57.91 (30). Nonetheless, these Ethiopian studies reported significantly lower mean QoL summary scores compared to a study conducted in Sweden. The Swedish study found the mean summary QoL score for cancer patients to be 81.4 (31).

In this study, the highest functional status was emotional functioning, with a mean score of 53.2. Though the score in this study was much lower, a similar study carried out in Gondar, Ethiopia, also reported that the highest functional status was emotional functioning, with a mean score of 61.0 (18). The lowest functional status in the current study was social functioning, with a mean score of 21.5. In contrast to the result of this research, in a study conducted in Addis Ababa, Ethiopia, role functioning was the lowest with a mean score of 23.8, and the highest was observed in social functioning with a mean score of 75.5 (32).

Among the symptom scales, the highest mean score was 83.3, which was attributed to dyspnea. However, our study revealed a significantly higher prevalence of dyspnea compared to a similar study conducted in Mumbai, India, which reported a prevalence rate of 44.37% in advanced cancer patients (33). The lowest mean score was reported for diarrhea, which was 32.8, though the majority of participants (65.5%) reported above this mean score. Similarly, a study carried out at the Tikur Anbessa Specialized Hospital in Addis Ababa, Ethiopia, found that among cervical cancer patients, diarrhea was one of the least reported symptoms (34). In this study, the mean score for financial impact was 82.6, significantly higher than the mean score of 54.1 for financial difficulties reported in a similar study conducted in Vietnam (35).

This study found that only 39.3% of the participants had a good QoL summary score. Most (60.7%) of the participants had been affected by cancer or its symptoms. The participants who had good functional QoL were 42.6%, which was slightly lower than a study conducted in Gondar, Ethiopia, which reported that 44.8% of the participants had a good functional QoL score (19). The participants who had good overall health status and overall QoL were 54.8% and 31.7%, respectively. The most affected functional score was social functioning, where only 35.5% of the participants had good social functioning, while the least affected social functioning was physical functioning, where 49.2% of the participants had good physical functioning. However, another study conducted in Gondar, Ethiopia, reported that the highest functional status was emotional functioning (18). Though the reported mean score for it was the lowest (32.8) among the symptom scales, the most common symptom among participants was diarrhea, affecting 65.5%, while appetite loss was the least prevalent at 34.3%. However, another study in Gondar, Ethiopia found appetite loss to be the most common symptom, impacting 77.1% of participants (19).

According to this study, patients with higher levels of education were more likely to have a better QoL compared to those who were illiterate. Specifically, patients with secondary school education were 3.16 times more likely to have a better QoL, while those with a diploma or higher-level education were 4.90 times more likely. This finding aligns with a study conducted among cancer survivors at a tertiary care cancer center in Malaysia, which reported that patients with a higher level of education exhibited an improved QoL (36). Indeed, research indicates that higher levels of college education are associated with a decreased likelihood of death from cancer (37).

Regarding occupational status, those patients working as civil servants were found to be 87% less likely to have a good QoL compared to farmers. This finding was supported by a Malaysian study, which showed that the most affected socio-demographic factor was employment (36). This could be attributed to an elevated level of job stress, potentially resulting from the negative impacts of cancer treatment like fatigue, pain, depression, and anxiety (38).

Concerning the place of residence, cancer patients living in urban areas were found to have a better QoL. They were 1.74 times more likely to have a higher QoL compared to those living in rural areas, which was also reported by a similar study in that QoL among cancer survivors in rural areas was poorer than that among urban cancer survivors (39). Another study conducted on non-Hodgkin’s lymphoma survivors also showed that rural residence was independently associated with lower physical functioning (40). This might be due to the typically fewer primary and specialty care physicians, as well as the limited number of home- and community-based service providers in rural areas compared to urban ones (41).

Another important factor was the stage of cancer, which was found to be a significant factor in QoL, with participants with stage III and IV cancer being 86% and 84% less likely, respectively, to have a good QoL compared to those with stage I cancer. This report is supported by a study from Bangladesh, indicating that functional scales decline while dyspnea and insomnia worsen as the cancer advances (42). In fact, as cancer progresses, the patients’ condition typically deteriorates due to factors such as tumor growth, metastasis, and the side effects of cancer treatments (43).

### Limitations

Since the study design was cross-sectional, it might have several limitations, including the inability to determine the exact direction of the relationship or influence of one variable on another. The study excluded cancer patients not undergoing chemotherapy, those receiving treatment at private hospitals, and those receiving treatments other than chemotherapy. Thus, the sample may not be generalizable to all patients with cancer and/or those receiving chemotherapy at private health facilities.

### Practical implication

The study highlights the importance of education and occupational health support in determining QoL for cancer patients. It suggests that higher levels of education can lead to better health outcomes and that certain occupations may be associated with lower QoL. Indeed, health literacy is closely linked with patients’ ability to engage in complex disease management and self-care (44). It might also be important to minimize the rural-urban disparities in cancer management and care (41). Moreover, the study emphasizes the negative impact of advanced cancer stages on QoL, indicating that early detection and treatment may be crucial for improving patient outcomes (45).

## Conclusion

The QoL of cancer patients in this study was found to be low, with lower functioning and a higher symptomatic and financial impact. These issues were also firmly expressed by the participants in the qualitative approach. The QoL of the patients was significantly associated with sociodemographic variables such as level of education, occupational status, area of residence, and the stage of cancer. In considering the concerning nature of the issue, psychosocial support seems indispensable.

## Ethics approval and consent to participate

Ethical approval was obtained from the research and community service office of DCSH. The study’s purpose was thoroughly explained to all participants, from whom informed consent was obtained. Participants who were literate put their signatures, and those who were illiterate affixed their fingerprints after the interviewer (MGY) verbally and briefly explained the purpose of the study to them and asked for their consent to participate in the study. Participant anonymity was maintained, and all data collected remained confidential. We also complied with the “World Medical Association Declaration of Helsinki: ethical principles for medical research involving human subjects” (46).

## Data availability statement

The data that support the findings of this study are available within the article.

## Ethics statement

The studies involving humans were approved by research and community service office of DCSH. The studies were conducted in accordance with the local legislation and institutional requirements. The participants provided their written informed consent to participate in this study.

## Author contributions

EB: Conceptualization, Data curation, Formal analysis, Investigation, Methodology, Project administration, Resources, Software, Supervision, Validation, Visualization, Writing – original draft, Writing – review & editing. MY: Conceptualization, Data curation, Formal analysis, Investigation, Methodology, Project administration, Resources, Software, Supervision, Validation, Visualization, Writing – original draft, Writing – review & editing. ZY: Conceptualization, Investigation, Methodology, Supervision, Writing – original draft, Writing – review & editing. HT: Conceptualization, Investigation, Methodology, Supervision, Writing – original draft, Writing – review & editing. SZ: Conceptualization, Investigation, Methodology, Supervision, Writing – original draft, Writing – review & editing.
