# Correction: Quality of life and associated factors among patients with cancer receiving chemotherapy at Dessie Comprehensive Specialized Hospital, North-East Ethiopia: a cross-sectional study

**DOI:** 10.3389/fonc.2025.1641735

**Published:** 2025-07-11

**Authors:** Ewunetie Mekashaw Bayked, Mekdes Getachew Yimam, Zemen Mengesha Yalew, Husien Nurahmed Toleha, Segenet Zewdie

**Affiliations:** ^1^ Department of Pharmacy, College of Medicine and Health Sciences (CMHS), Wollo University, Dessie, Ethiopia; ^2^ Department of Oncology, Dessie Comprehensive Specialized Hospital (DCSH), Dessie, Ethiopia; ^3^ Department of Comprehensive Nursing, College of Medicine and Health Sciences (CMHS), Wollo University, Dessie, Ethiopia; ^4^ Department of Pharmacy, College of Medicine and Health Science, Injibara University, Injibara, Ethiopia

**Keywords:** cancer, patients, chemotherapy, quality of life, associated factors

In the published article, under the methods subsection, i.e., data processing and analysis, “**Equation 6**” is not correct, which was:


$$“Mean\;QLQ_{C30}\;summary\;score=\;\frac{\sum \left(Functional\;scales\right)-\sum (100-symptom\;scales)”}{13}$$

The error happened because we accidentally used the “minus” sign instead of the “plus” sign, although we applied the correct computation to calculate the mean QLQ_C30 summary score.

The correct formula is:


(6) $$“Mean\;QLQ_{C30}\;summary\;score=\;\frac{\sum \left(Functional\;scales\right)\;+\;\sum (100-symptom\;scales)”}{13}$$

The original version of this article has been updated.

